# Correction: Toward realistic computer modeling of paraffin-based composite materials: critical assessment of atomic-scale models of paraffins

**DOI:** 10.1039/d0ra90087g

**Published:** 2020-08-24

**Authors:** Igor V. Volgin, Artyom D. Glova, Victor M. Nazarychev, Sergey V. Larin, Sergey V. Lyulin, Andrey A. Gurtovenko

**Affiliations:** Institute of Macromolecular Compounds, Russian Academy of Sciences Bolshoi Prospect V.O. 31 St. Petersburg 199004 Russia a.gurtovenko@gmail.com

## Abstract

Correction for ‘Toward realistic computer modeling of paraffin-based composite materials: critical assessment of atomic-scale models of paraffins’ by Artyom D. Glova *et al.*, *RSC Adv.*, 2019, **9**, 38834–38847, DOI: 10.1039/C9RA07325F.

The authors regret that the 1–4 Lennard-Jones interactions were not accounted for in the molecular dynamics simulations of *n*-eicosane samples with the use of the united-atom GROMOS force field, as required by the original parametrization of this force field.^1^ This led to the abnormal behavior of the corresponding systems, namely: the *n*-eicosane samples did not crystallize within the temperature range of 200–450 K. After the 1–4 interactions were turned on, the GROMOS force field allowed us to observe the crystallization of *n*-eicosane with the transition temperature of 270 ± 1 K, see Fig. 1.

**Fig. 1:**
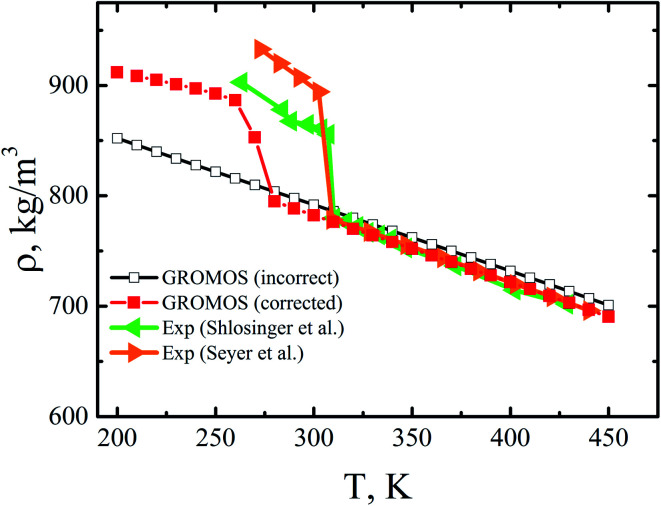
Mass density of *n*-eicosane samples as a function of temperature for the GROMOS force field, *cf.* Fig. 2(b) of the original publication. The open black squares show the original incorrect data, and the closed red squares correspond to the corrected results. For the sake of comparison, the experimental data^2,3^ is also presented.

Most structural and dynamic characteristics also changed after the 1–4 interactions were properly accounted for. Below we present the updated versions of Tables 1 and 2 of the original publication.

**Table 1:** The crystallization temperature (*T*_c_), the coefficient of volumetric thermal expansion (CTE) measured in the temperature interval 400–450 K, the radius of gyration (*R*_g_), and the end-to-end distance *H* of *n*-eicosane chains for different force fields. The corrected data for the GROMOS force field is highlighted in bold

Force field	*T*_c_, K	CTE, 10^−4^ K^−1^	*R*_g_, nm (*T* = 250 K)	*R*_g_, nm (*T* = 450 K)	*H*, nm (*T* = 250 K)	*H*, nm (*T* = 450 K)
GAFF	330 ± 1	17.4 ± 0.1	0.75 ± 0.01	0.60 ± 0.01	2.43 ± 0.01	1.70 ± 0.01
GAFF2	365 ± 2	14.3 ± 0.1	0.75 ± 0.01	0.62 ± 0.01	2.43 ± 0.01	1.82 ± 0.01
OPLS-AA	365 ± 1	14.7 ± 0.1	0.74 ± 0.01	0.62 ± 0.01	2.42 ± 0.01	1.80 ± 0.01
L-OPLS-AA	265 ± 3	12.3 ± 0.1	0.72 ± 0.01	0.57 ± 0.01	2.29 ± 0.01	1.63 ± 0.01
CHARMM36	320 ± 1	12.4 ± 0.1	0.74 ± 0.01	0.59 ± 0.01	2.39 ± 0.01	1.69 ± 0.01
**GROMOS**	**270 ± 1**	**9.1 ± 0.1**	**0.72 ± 0.01**	**0.58 ± 0.01**	**2.34 ± 0.01**	**1.66 ± 0.01**
NERD	270 ± 1	11.6 ± 0.1	0.74 ± 0.01	0.58 ± 0.01	2.41 ± 0.01	1.64 ± 0.01
OPLS-UA	310 ± 1	7.6 ± 0.1	0.73 ± 0.01	0.57 ± 0.01	2.38 ± 0.01	1.63 ± 0.01
PYS	270 ± 10	9.0 ± 0.1	0.72 ± 0.01	0.55 ± 0.01	2.34 ± 0.01	1.57 ± 0.01
TraPPE	280 ± 1	9.6 ± 0.1	0.74 ± 0.01	0.58 ± 0.01	2.41 ± 0.01	1.65 ± 0.01
Experiment	310 (ref. 4)	8.8–8.9 (ref. 2 and 3)	—	—	2.43 (ref. 5)	—

**Table 2:** The shear viscosity, the diffusion coefficient and the mass density for *n*-eicosane samples in the liquid state simulated with different force fields at *T* = 450 K. The corrected data for the GROMOS force field is highlighted in bold

Force field	*η*, mPa s	*D*, 10^−5^ cm^2^ s^−1^	*ρ*, kg m^−3^
GAFF	0.42 ± 0.04	2.9 ± 0.2	592.3 ± 0.1
GAFF2	0.59 ± 0.01	2.1 ± 0.1	633.5 ± 0.1
OPLS-AA	0.83 ± 0.07	1.6 ± 0.1	668.2 ± 0.1
L-OPLS-AA	0.64 ± 0.08	2.2 ± 0.2	656.4 ± 0.1
CHARMM36	0.60 ± 0.04	2.1 ± 0.2	658.1 ± 0.1
**GROMOS**	**0.52 ± 0.01**	**2.7 ± 0.1**	**690.3 ± 0.1**
NERD	0.32 ± 0.01	3.6 ± 0.1	661.0 ± 0.1
OPLS-UA	0.79 ± 0.02	1.8 ± 0.1	753.1 ± 0.1
PYS	0.53 ± 0.01	2.6 ± 0.1	698.8 ± 0.1
TraPPE	0.45 ± 0.04	2.9 ± 0.1	693.7 ± 0.1
Experiment	0.594 (*T* = 453 K)^6^	2.2 (*T* = 443 K)^7^	696.0 (*T* = 440 K)^3^

Overall, the GROMOS force field does not show abnormal behavior for the *n*-eicosane sample anymore and performs rather similarly to other united-atom force fields such as *e.g.* PYS. The main conclusions of the original publication remain unchanged since the use of all-atom general-purpose force fields was shown to provide a more realistic description for *n*-eicosane as compared to their united-atom counterparts.

The Royal Society of Chemistry apologises for these errors and any consequent inconvenience to authors and readers.

## Supplementary Material
